# A novel blood-based epigenetic biosignature in first-episode schizophrenia patients through automated machine learning

**DOI:** 10.1038/s41398-024-02946-4

**Published:** 2024-06-17

**Authors:** Makrina Karaglani, Agorastos Agorastos, Maria Panagopoulou, Eleni Parlapani, Panagiotis Athanasis, Panagiotis Bitsios, Konstantina Tzitzikou, Theodosis Theodosiou, Ioannis Iliopoulos, Vasilios-Panteleimon Bozikas, Ekaterini Chatzaki

**Affiliations:** 1 Laboratory of Pharmacology, Department of Medicine, Democritus University of Thrace, GR-68132 Alexandroupolis, Greece; 2 Institute of Agri-food and Life Sciences, University Research & Innovation Center, H.M.U.R.I.C., Hellenic Mediterranean University, GR-71003 Crete, Greece; 3 II. Department of Psychiatry, Faculty of Health Sciences, School of Medicine, Aristotle University of Thessaloniki, GR-56430 Thessaloniki, Greece; 4 Ι. Department of Psychiatry, Faculty of Health Sciences, School of Medicine, Aristotle University of Thessaloniki, GR-56429 Thessaloniki, Greece; 5 Department of Psychiatry and Behavioral Sciences, Faculty of Medicine, University of Crete, GR-71500 Heraklion, Greece; 6 ABCureD P.C, GR-68131 Alexandroupolis, Greece; 7 Division of Basic Sciences, School of Medicine, University of Crete, GR-71003 Heraklion, Greece; 8 Institute of Molecular Biology and Biotechnology, Foundation for Research and Technology, 70013 Heraklion, Greece

**Keywords:** Diagnostic markers, Schizophrenia, Predictive markers

## Abstract

Schizophrenia (SCZ) is a chronic, severe, and complex psychiatric disorder that affects all aspects of personal functioning. While SCZ has a very strong biological component, there are still no objective diagnostic tests. Lately, special attention has been given to epigenetic biomarkers in SCZ. In this study, we introduce a three-step, automated machine learning (AutoML)-based, data-driven, biomarker discovery pipeline approach, using genome-wide DNA methylation datasets and laboratory validation, to deliver a highly performing, blood-based epigenetic biosignature of diagnostic clinical value in SCZ. Publicly available blood methylomes from SCZ patients and healthy individuals were analyzed via AutoML, to identify SCZ-specific biomarkers. The methylation of the identified genes was then analyzed by targeted qMSP assays in blood gDNA of 30 first-episode drug-naïve SCZ patients and 30 healthy controls (CTRL). Finally, AutoML was used to produce an optimized disease-specific biosignature based on patient methylation data combined with demographics. AutoML identified a SCZ-specific set of novel gene methylation biomarkers including *IGF2BP1, CENPI*, and *PSME4*. Functional analysis investigated correlations with SCZ pathology. Methylation levels of *IGF2BP1* and *PSME4*, but not *CENPI* were found to differ, *IGF2BP1* being higher and *PSME4* lower in the SCZ group as compared to the CTRL group. Additional AutoML classification analysis of our experimental patient data led to a five-feature biosignature including all three genes, as well as age and sex, that discriminated SCZ patients from healthy individuals [AUC 0.755 (0.636, 0.862) and average precision 0.758 (0.690, 0.825)]. In conclusion, this three-step pipeline enabled the discovery of three novel genes and an epigenetic biosignature bearing potential value as promising SCZ blood-based diagnostics.

## Introduction

Schizophrenia (SCZ) is a chronic, severe, and debilitating psychiatric disorder with a complex and heterogeneous genetic and neurobiological background, that influences early brain development and affects all areas of personal functioning [1–4]. The disorder is typically characterized by “positive” (e.g., delusions, hallucinations, disorganized behavior, and thinking), “negative” (e.g., loss of motivation and interest, social withdrawal, anhedonia, affective flattening), and “cognitive” symptoms and is associated with serious disability and functional impairment, as well as with a much higher risk of physical and mental comorbidities and a much lower overall life expectancy [1–4].

The pathophysiology of SCZ is extremely complex and, despite the qualitative research of the past decades, only in part understood. The neurodevelopmental hypothesis of SCZ postulates that the risk of developing the disorder lies in a heritable risk and additional environmental exposures that occur throughout development [5, 6]. Accordingly, although SCZ features a very strong genetic component with a heritability of about 80%, a very broad range of additional environmental factors and stressors has been suggested to contribute to the neurodevelopment of the disorder. Hence, only 50% of monozygotic twins of patients develop the disease, pointing to a significant role of epigenetic modifications. Such epigenetic modifications include DNA methylation, histone modifications, and non-coding RNAs [7]. Accumulating knowledge suggests that altered DNA methylation profiles of several genes are implicated in the pathogenesis of SCZ and related psychiatric disorders. For example, studies on the reelin gene (*RELN*), an important GABAergic candidate gene, revealed increased methylation levels of *RELN* promoter in different brain areas of patients with SCZ [8] and the peripheral blood of SCZ patients [9]. Regarding the dopaminergic pathway, methylation studies have focused on the catechol-O-methyltransferase gene (*COMT*). In an epigenetic analysis of post-mortem human brains, authors found significant hypomethylation of *COMT* promoter in SCZ patients [10] also noted in the saliva DNA of SCZ patients [11]. Methylation analysis of the serotonin signaling receptor genes, 5-hydroxytryptamine receptor 1 A (*HTR1A*) and 5-hydroxytryptamine receptor 2 A (*HTR2A*) revealed significant associations between hypermethylation and SCZ brain [12] or blood [13]. Furthermore, multiple studies have shown that blood DNA methylation of the brain-derived neurotrophic factor gene (*BDNF*) is related to SCZ [14, 15], although altered *BDNF* methylation was not observed in brain tissue [16].

Among many others, urbanicity, viral infections, maternal immune activation, obstetric complications, nutrient deprivation, parental absence, toxins, childhood trauma, cannabis exposure, etc. have been all repeatedly shown to be related to a higher risk of developing SCZ [1–4]. The long-lasting impact of such environmental factors and stressors on neurodevelopment is suggested to be mainly mediated by epigenetic modifications that alter genome function [6].

Although there is mounting evidence for several functional, neuroanatomical, and molecular alternations in SCZ, the routine diagnosis and treatment follow-up of the disorder still mainly depends on clinical examination, psychometric scales, and medical history. This fact underlines the urgent need for new reliable, routinely applicable, and easily accessible objective biomarkers for the early diagnosis and treatment of SCZ [17, 18]. However, despite the increasing research interest in biomarkers in psychiatry, very few have been established in clinical practice and many findings remain unconfirmed [19]. Lately, special attention has been given to blood-based, epigenetic biomarkers in SCZ [20].

However, single biomarkers are unlikely to bear the accuracy and validity required to become clinically relevant. Instead, genome-wide methylation analyses through microarrays or next-generation sequencing (NGS) enable the study of a vast number of methylation-relevant regions of DNA where a cytosine nucleotide is followed by a guanine nucleotide in the linear sequence of bases along its 5’ → 3’ direction (CpG sites), providing high-dimensional datasets. Machine learning (ML) techniques for their analysis offer the opportunity to build biosignatures (panels of biomarkers) of personalized clinical importance [21–23]. Moreover, ML automation through highly innovative tools (AutoML) is increasingly popular, as it enables deep exploitation of omics datasets by applying multiple algorithms and performing effective feature selection. Automated machine learning (AutoML) is a process that allows to fully automate the machine learning process end-to-end. In particular, the JADBio platform used in this study, is an ad-hoc tool for biomedical research, with a proven capacity to effectively build predictive models by analyzing relatively small datasets, such as often those of patients [24–31]. It can automate the pre-analysis steps including data integration, preprocessing, cleaning, and engineering (feature construction), the analysis steps including algorithm selection, training of the models, and hyperparameter optimization, as well as the post-analysis steps including interpretation, explanation, and visualization of the analysis process and the output model. The main advantages of AutoML tools lie upon no requirement for coding knowledge, reduction of analysis time, and minimization of human-caused mistakes. Our research group has already published important AutoML-driven results and predictive models in COVID-19 [32], diabetes [33], Alzheimer’s disease [34], breast cancer [35], and suicide risk amongst depressive patients [36].

In this study, we have used a three-step, AutoML-based, data-driven biomarker discovery pipeline approach, using data from genome-wide DNA methylation datasets to build specific biosignatures, followed by pilot clinical validation via methylation analysis in the blood of SCZ patients and healthy controls. We deliver highly performing, blood-based epigenetic biosignatures that may hold promise for clinically relevant interventions and could promote the understanding of disease pathophysiology.

## Subjects and methods

**Fig. 1:**
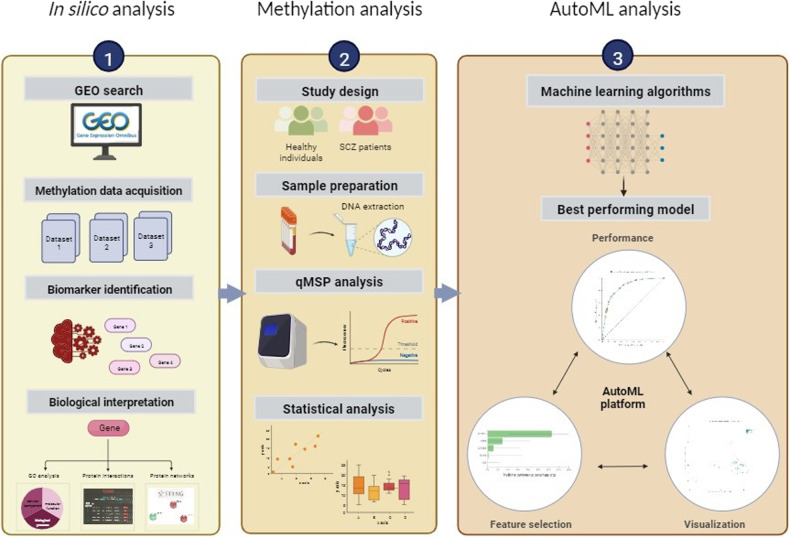
Workflow of our study. Our study uses a three-step approach to discover, validate and deliver a highly performing, blood-based epigenetic biosignature of diagnostic clinical value in SCZ. Step 1 - In silico analysis: At first, The Gene Expression Omnibus (GEO) database was used to retrieve publicly available, blood-based DNA methylation data from SCZ patients and healthy individuals. Following, feature selection was performed via AutoML to identify a minimum subset of features bearing the maximal classifying ability between groups. Identified features/genes were further investigated for their biological relevance to SCZ including gene ontology and pathway analysis using the GeneCards database, the identification of functional relationships with other known protein-coding genes related to the SCZ using the multi-UniReD tool, and the identification of possible protein-protein interaction networks using the STRING database. Step 2 - Methylation Analysis: The methylation profiles of the identified genes were investigated using the SYBR green-based qMSP method in blood-derived gDNA samples of 30 first-onset, drug-naive SCZ patients compared to 30 healthy individuals. Step 3 - AutoML Analysis: In our AutoML classification analysis, we employed the AutoML technology JADBio to produce a best-performing disease-specific biosignature based on our experimental patient methylation data combined with demographic data. Abbreviations: GEO Gene Expression Omnibus, SCZ Schizophrenia, qMSP quantitative Methylation Specific PCR, AutoML Automated Machine Learning.

This study uses a three-step approach to discover, validate, and deliver a highly performing, blood-based epigenetic biosignature of diagnostic clinical value in SCZ (*cf*. Figure 1). Overall, our pipeline focuses on the exploitation and knowledge mining of high-throughput methylome datasets to establish specific disease biosignatures to be validated in clinical practice and promote the understanding of SCZ pathophysiology.

### Step 1—in silico analysis

Through an AutoML-aided, data-driven biomarker discovery approach using data from high-throughput microarray blood DNA methylome datasets, we first identified SCZ-specific gene methylation biomarkers. Genes identified were further investigated for their biological relevance to known SCZ pathophysiological pathways.

#### Data sources

The Gene Expression Omnibus (GEO) database [37] was used to retrieve publicly available, blood-based DNA methylation data from SCZ patients and healthy individuals. The GEO database was searched using “*Schizophrenia*” as a keyword, “*Methylation profiling by array*” as the study type, and “*Homo sapiens”* as the organism of interest. In total, 19 studies were found; between them, only those using the Infinium Human Methylation 27k, 450k, or 850k BeadChips arrays, blood as the study tissue, and providing adequate normalized data were selected for further analysis, in particular three studies, namely GSE41037 [38], GSE157252 [39], and GSE41169 [38]. Dataset information is presented in Table 1.

**Table 1:** Performance overview, features, selection method and predictive algorithm of AutoML produced models of separate methylome datasets used for in silico analysis.

Dataset	Illumina Infinium Platform BeadChip	Study groups	Ref.	Gene features	Feature selection method	Predictive algorithm	Accuracy	Precision	AUC	JADBio analysis link
**GSE41037**	27k	394 CTRL 325 SCZ	[38]	CENPI IGF2BP1 PSME4	SES	SVM	0.743 [0.712, 0.795]	0.817 [0.757, 0.867]	0.836 [0.791, 0.875]	https://app.jadbio.com/share/bc38a8a0-b7c7-4c08-bd04-8433c1b14cc3
**GSE41169**	450k	33 CTRL 62 SCZ	[38]	five 3-feature models	SES	SVM	0.654 [0.597, 0.708]	0.673 [0.574, 0.686]	0.502 [0.397, 0.611]	https://app.jadbio.com/share/bc38a8a0-b7c7-4c08-bd04-8433c1b14cc3
**GSE157252**	850k	50 CTRL 21 SCZ	[39]	MDGA1 CISD3	LASSO	SVM	0.831 [0.759, 0.900]	0.898 [0.842, 0.95]	0.870 [0.790, 0.936]	https://app.jadbio.com/share/3f1c6b93-49f9-4e3f-864a-777b4d1c7309

Genome-wide methylation microarray data from the abovementioned studies were retrieved from GEO and analyzed using the AutoML platform JADBio to build SCZ-specific methylation biosignatures in blood. GSE41037 dataset consisted of 27,578, GSE41169 dataset of 485,577, and GSE157252 dataset of 865,432 CpG gene array features. Given a 2D matrix of data, JADBio automatically preprocesses data, performs feature selection by employing LASSO or SES algorithms, tries several algorithms and thousands of algorithmic configurations, and then selects the best set of features (biosignature), estimates the out-of-sample model’s performance after bootstrap correction and cross-validation, and provides several visualizations. A 2D matrix of each study consisting of methylation normalized beta values ranging between 0.0 (no methylation) and 1.0 (full methylation) for each CpG site of the Illumina Beadchips and the demographical data of each subject was uploaded to JADBio. Analyses of datasets produced gene-based models via different predictive algorithms reaching different accuracy, precision, and AUC. All analyses can be found in the respective JADBio links. Abbreviations: *AUC* Area Under the Curve, *SES* test-budgeted Statistically Equivalent Signature, *LASSO* Least Absolute Shrinkage, and Selection Operator, *SVM* Support Vector Machines.

#### Biomarker identification

Feature selection performed via AutoML identifies a minimum subset of features bearing the maximal classifying ability between groups. The innovative and specially designed for analyzing high-dimensional biological datasets AutoML technology JADBio, version 1.2.8 [24], was employed to build SCZ-specific biosignatures based on the retrieved high-dimensional methylation data and the demographical information as previously described [32, 34]. JADBio applies to low or high-sample data, as well as to high-dimensional or low-scale omics data, and produces accurate predictive models estimating the out-of-sample model’s performance after bootstrap correction and cross-validation. JADBio preprocesses data including mean imputation, mode imputation, constant removal, and standardization, and then, tries several predictive algorithms such as Classification Random Forests, Support Vector Machines, Ridge Logistic Regression, and Classification Decision Trees. Specifically, for small sample sizes, it employs a stratified, K-fold, repeated cross-validation BBC-CV algorithm protocol that exhibits small estimation variance and removes estimation bias. BBC-CV’s main idea is to bootstrap the whole process of selecting the best-performing configuration on the out-of-sample predictions of each configuration, without additional training of models [25].

For each AutoML analysis, we used extensive model tuning effort, we chose the area under the curve (AUC) metric for optimization of biosignature performance and we set the classifier maximum size to three features (*cf*. Table 1, legend). The predictive power of each biosignature was assessed using AUC and average precision (also known as area under the precision-recall curve) metrics.

#### Biological interpretation

To explore the CpG sites included in the specific biosignatures built by AutoML, each CpG was allocated to its corresponding gene with an extended window size of 20 kb downstream and upstream. Identified genes and respective proteins were further studied to unfold relevance to SCZ pathology. Gene Ontology (GO) and pathway data for each gene were retrieved via the GeneCards Suite [40]. The GO analysis covers three domains: molecular function, the elemental activities of a gene product at the molecular level; cellular component, the parts of a cell or its extracellular environment; biological process, chemical reactions, or other events that are involved and are pertinent to the functioning of integrated living units: cells, tissues, organs, and organisms. Also, subcellular location and tissue expression distribution were retrieved via the UniProtKB database information [41].

Then, we employed the *multiple* UniReD tool [42, 43] to identify the functional relationships of respective proteins. *Multiple* UniReD is a mining tool of published biomedical literature that associates the proteins of interest (query list) to a list of reference proteins that are known and verified to be involved in the disease under investigation (reference list) [44–46]. *Multiple* UniReD produces a score for each protein of interest that signifies its relatedness to the proteins in the reference list. The higher the score the higher the functional association of the proteins of interest to the reference list proteins. In particular, *multiple* UniReD searches for the existence of any association in UniReD clusters between the protein pairs of the two lists (reference and query list). If the pair is present in a UniReD cluster, the association will obtain a score of 1 and UniReD will continue to the next pair of reference and query proteins. If a protein has not been analyzed by UniReD, no results will be retrieved. If *multiple* UniReD cannot find any association within the clusters, it will proceed to the next step in order to search for paralogues. More specifically, *multiple* UniReD will search for a co-occurrence of a paralogue of the query protein with the reference protein in a UniReD cluster. If such a pair is present, the association will obtain a score of 0.5. In the case that UniReD does not predict any association within a query paralogue and the reference protein, then multiple UniReD will investigate whether the query protein is part of a complex. In this case, it will search for co-occurrences of the reference protein in the complex. If such an association is confirmed, the association will obtain a score of 0.5. Finally, if none of the above applies, UniReD will search for a relation between the orthologues of reference and query proteins. If such an association is documented, it will obtain a score of 0.5. When none of the aforementioned cases are applicable, then no score will be assigned to the specific protein pair [43]. In order to build our reference list of genes with a known and established role in SCZ and to include an adequate number of genes to cover multiple pathways, we searched the literature for the most cited candidate genes and we were also based on descriptive genetic reviews [47–51]. We then performed a further focused study for each identified protein. A 33 list of protein-coding genes known for their implication in SCZ pathophysiology according to the literature was built and used in *multiple* UniReD, to test the associations of identified features. Reference genes used are presented in Suppl. Table S1.

Finally, in an attempt to identify possible protein-protein interaction networks among features and the 33 protein-coding genes, we performed a functional protein association network analysis using the STRING database [52]. STRING is a database of known and predicted protein-protein interactions. The interactions include direct (physical) and indirect (functional) associations; they stem from computational prediction, knowledge transfer between organisms, and interactions aggregated from other (primary) databases.

### Step 2—methylation analysis

Following the in silico analysis, the methylation profiles of the selected genes were investigated using the SYBR green-based methylation-specific polymerase chain reaction (qMSP) method in blood-derived genomic DNA (gDNA) samples of SCZ patients compared to healthy individuals, in order to validate the initial biosignatures in a laboratory setting and their clinical validity. Finally, further AutoML analysis of methylation and clinical data led to optimized diagnostic biosignatures of clinical importance.

#### Clinical Samples

Our study’s groups consisted of 30 first-onset, drug-naive SCZ patients and 30 age- and sex-matched healthy individuals (control group, CTRL) without other mental or physical comorbidities. Screening included a thorough physical and neurological examination, routine blood laboratory tests, urine toxicology screen, electrocardiogram (ECG), and a structured face-to-face clinical interview. Inclusion criteria of SCZ patients included: age 18 - 45 years, first episode, drug naïve, being able to sign informed consent and diagnosis of schizophrenia, schizoaffective disorder, schizophreniform disorder, brief psychotic disorder, or psychotic disorder not otherwise specified confirmed individually by two separate experienced psychiatrists according to the diagnostic criteria of the fourth edition of the Diagnostic and Statistical Manual of Mental Disorder-IV Text Revision (DSM-IV-TR) and the Mini International Neuropsychiatric Interview plus. The operational criteria for the first episode were: first episode of uninterrupted positive symptoms, no matter the duration, no symptom remission for 1 month or longer duration. Exclusion criteria included the presence or self-reported history of any chronic or acute physical and Axis I mental co-morbidities, body mass index (BMI) values beyond 18-30 kg/m2, frequent usage of any either illicit or prescribed drugs or over-the-counter medications, drinking of more than 100 g of alcohol per week, abnormal physical and neurological examinations, basic blood laboratory test values deviating from the normal range, positive urine toxicology screen, pregnancy, nursing, and pathological initial ECG. Hypothyroidism in the euthyroid state through hormonal substitution, as well as hypertension in the normotensive state through antihypertensive medication, did not serve as exclusion criteria. SCZ patients were recruited in the Dept. of Psychiatry at the Aristotle University of Thessaloniki. Psychopathology symptom severity of SCZ patients was evaluated with the Positive and Negative Syndrome Scale (PANSS). Age- and sex-matched healthy control individuals were recruited from the blood donation unit of the University General Hospital of Alexandroupolis using the same exclusion criteria. Demographic and psychometric data of both SCZ and CRTL groups are presented in Suppl. Table S2. The study was approved by the Ethics Review Committee (ERC) of the Aristotle University of Thessaloniki and the University General Hospital of Alexandroupolis, Greece, and was conducted according to the ethical principles of the 1964 Declaration of Helsinki and its later amendments. After a full oral and written explanation of the purpose and procedures of the investigation, written informed consent was obtained from each patient and healthy control participant before initiating the screening procedure and enrollment in the study.

#### qMSP analysis

Methylation analysis was performed in 30 SCZ samples and 30 CTRL samples. Blood samples were obtained in EDTA-coated tubes between 09.00 and 12.00 of each study day. Blood was stored at −20 °C until further analysis. Genomic DNA (gDNA) from peripheral blood was extracted using the QIAamp DNA Blood Mini kit (Qiagen, Germany) according to the manufacturer’s instructions. Then, the gDNA quantity was checked via Nanodrop Spectrophotometer (Thermo Fisher Scientific, UK) and then was stored at −20 °C. Bisulfite conversion of DNA was performed by EZ DNA Methylation-Gold ™ Kit (ZYMO Research, USA) as suggested by the manufacturer. In each reaction, CpGenome Human methylated and non-methylated DNA controls (Merck Millipore, Germany) were included as negative and positive control samples, respectively. The converted gDNA was stored at −80 °C, ready for methylation analysis.

A methylation-independent PCR assay for the β-actin gene (*ACTB*) was used to verify the sufficient quality and quantity of converted gDNA. Methylation levels of investigated genes were analyzed using quantitative SYBR Green-based methylation-specific PCR (qMSP) assays. Primers specific for the methylated sequence of each gene, all found in the gene body, were newly designed using the MethPrimer software [53]. Primer sequences are provided in Suppl. Table S3. To set up robust qMSP assays, extensive optimization was performed. Specificity and cross-reactivity of primers were evaluated using unconverted gDNA and converted methylated and non-methylated DNA controls. The analytical specificity of qMSP assays was evaluated by using mixes of methylated and non-methylated DNA standards (100%, 50%, 10%, 1%, 0%). The analytical sensitivity of assays was evaluated using serial dilutions of methylated and non-methylated DNA controls in H_2_O. The reproducibility (calculated as coefficients of variation, CVs), efficiency, and linearity were also evaluated in order to complete the validation file of the established assays. All samples were run in duplicates. The results were calculated using the Rotor-Gene 6000 Series Software 1.7 (Qiagen). The analysis was performed according to the RQ sample (Relative Quantification) = 2^−∆∆CT^ method [54]. Specifically, ∆∆C_T_ values were generated for each target after normalization by ACTB values and using 100% methylated control as a calibrator.

### Statistical analysis

Initially, the Kolmogorov–Smirnov test was applied to check for normality in the distribution of continuous methylation data. Due to the lack of normality in our data, the Mann–Whitney *U* test and Spearman correlation test were used for pairwise comparisons between CTRL and SCZ groups. Continuous variables are expressed as median (minimum-maximum) or mean ± standard deviation (SD). Categorical variables are shown as absolute frequencies (percentages). In all tests performed, statistical significance was set at a two-sided *p*-value < .05. Statistical analysis was carried out with the jamovi version 2.3 statistical software package retrieved from https://www.jamovi.org.

### Step 3—AutoML analysis

For our AutoML classification analysis, we employed the AutoML technology JADBio [24] to produce optimized disease-specific biosignatures based on our experimental patient methylation data combined with demographic data (cf. Table 3, Legend). For the analysis, we used extensive model tuning effort and we chose the AUC metric for optimization of performance. The AUC is also used to assess the overall performance of the classification model.

## Results

### In silico-built schizophrenia-specific biosignatures

**Fig. 2:**
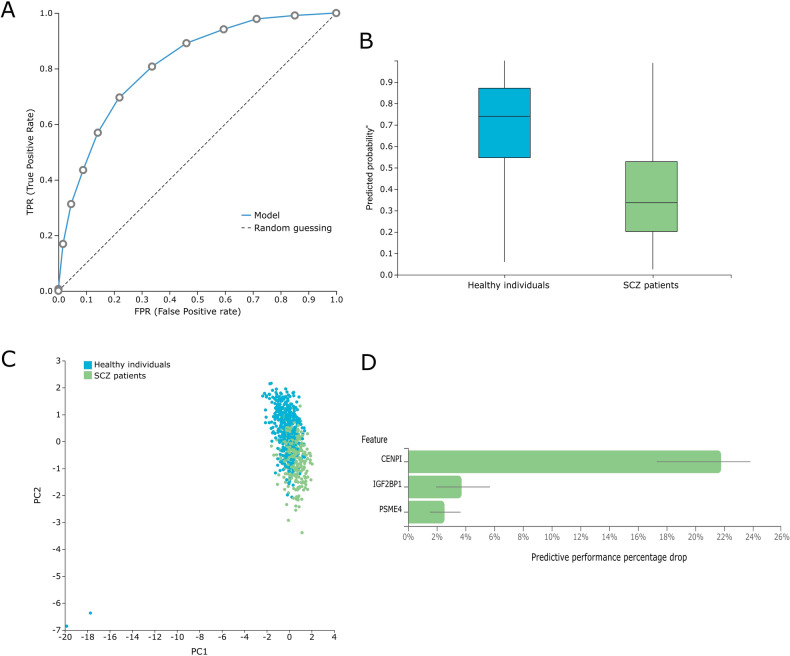
SCZ-specific methylation biosignature built from the GSE41037 dataset using AutoML. **A** ROC curve of the model. **B** Out-of-sample probability box plot (i.e., probability predictions when samples were not used for training). **C** PCA plot shows the separation between SCZ patients (green) and healthy individuals (blue). **D** Feature Importance plot of the features of the model. Feature importance is defined as the percentage drop in predictive performance when the feature is removed from the model. Abbreviations: ROC Receiver Operating Characteristic, PCA Principal Component Analysis.

Genome-wide blood methylation microarray data from the GSE41037, GSE41169, and GSE157252 studies retrieved from GEO were analyzed using the AutoML platform JADBio to build SCZ-specific methylation biosignature models. The methylation dataset is uploaded to the platform, and analysis is performed automatically by applying multiple machine learning algorithms and performing effective feature selection. The analysis steps include algorithm selection, training of the models, and hyperparameter optimization, as well as the post-analysis of the output model. The biosignature delivered includes a minimum subset of features bearing the maximal classifying ability between groups. The models’ performance metrics are calculated automatically, following internal validation. Features of each model and a performance overview as well as the feature selection method and the predictive algorithm are presented in Table 1. Among the three datasets, the GSE41037 dataset biosignature and its included biomarkers (https://app.jadbio.com/share/bc38a8a0-b7c7-4c08-bd04-8433c1b14cc3) were selected for further analysis and clinical validation in our clinical cohort due to the favorable combination of the larger study group size, the high performance demonstrated in silico (*cf*. Figure 2) and the greater score in functional associations to SCZ (see below).

### Biomarker biological interpretation

Genes featured in the GSE41037 and GSE157252 dataset biosignatures were found to be involved in several biological processes such as sex differentiation, centromere complex assembly, regulation of cytokine production, neuron migration, nervous system development, DNA repair, cellular response to DNA damage stimulus, and protein maturation by [2Fe-2S] cluster transfer (*cf*. Table 2). According to GeneCards, CENPI and PSME4’s molecular function is related to protein binding in the nucleus of the cell, while IGFBP1’s molecular function is associated with nucleic acid binding, also in the nucleus of the cell. CISD3’s is related to metal ion binding in the cytoplasm and mitochondria of the cell and MDGA1’s molecular function is associated with protein binding in the extracellular space.

**Table 2:** Featured genes in the GSE41037 and GSE157252 datasets AutoML SCZ-specific biosignatures and their biological characteristics and functions.

Gene	Gene description	Gene type	Pathway	GO-Molecular function	GO-Cellular component	GO-Biological process	Subcellular location	Tissue specificity
***IGF2BP1***	Insulin-Like Growth Factor 2 mRNA Binding Protein 1	Protein coding	Cytoskeletal signaling, Translational control, Wnt/ Hedgehog/ Notch	Enables nucleic acid binding	Active in nucleus	Involved in regulation of cytokine production and nervous system development	Localizes in nucleus, cytoplasm	Mainly expressed in the embryo. Also expressed in follicles of ovary, as well as in gonocytes of testis, and placenta
***PSME4***	Proteasome Activator Subunit 4	Protein coding	CDK-mediated phosphorylation and removal of Cdc6	Enables protein binding	Active in nucleus	Involved in DNA repair and cellular response to DNA damage stimulus	Localizes in cytoplasm, nucleus	Expressed in sperm and 205 other cell types or tissues
***CENPI***	Centromere Protein I	Protein coding	Cell cycle, mitotic, Chromosome maintenance	Enables protein binding	Active in nucleus	Involved in sex differentiation and centromere complex assembly	Localizes exclusively in the centromeres, in the nucleus	Expressed in secondary oocyte and 131 other cell types or tissues
***MDGA1***	MAM Domain Containing Glycosylphosphatidylinositol Anchor 1	Protein coding	Metabolism of proteins, post-translational modification: synthesis of GPI-anchored proteins	Enables protein binding	Active in extracellular space, plasma membrane	Involved in neuron migration and nervous system development	Localizes in cell membrane and is associated with lipid rafts.	Has been found in brain, heart, skeletal muscle and kidney. Found to be overexpressed in tumor tissues.
***CISD3***	CDGSH Iron Sulfur Domain 3	Protein coding	Iron-sulfur cluster binding	Enables metal ion binding	Active in mitochondria and cytoplasm	Involved in protein maturation by 2Fe-2S cluster transfer	Localizes in mitochondria	Expressed in mucosa of transverse colon and 97 other cell types or tissues

The genes featured in the GSE41037 and GSE157252 dataset biosignatures were subjected to pathway and Gene Ontology (GO) analysis using the GeneCards database. The GO analysis covers three domains: molecular function, the elemental activities of a gene product at the molecular level; cellular component, the parts of a cell or its extracellular environment; biological process, chemical reactions, or other events that are involved and are pertinent to the functioning of integrated living units: cells, tissues, organs, and organisms. Subcellular location and the tissue expression distribution are reported according to the UniprotKB database information.

**Fig. 3:**
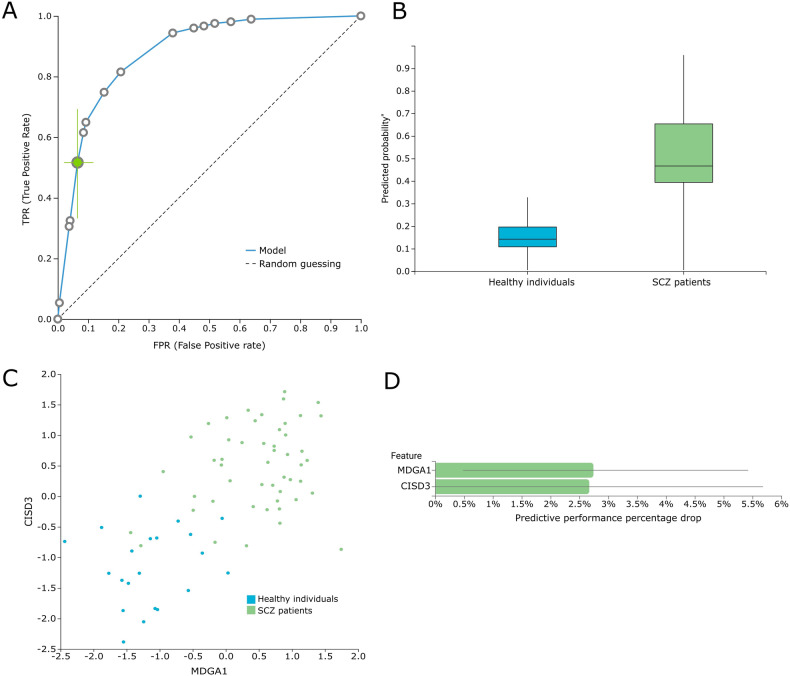
SCZ-specific methylation biosignature built from the GSE157252 dataset using AutoML. **A** ROC curve of the model. **B** Out-of-sample probability box plot (i.e., probability predictions when samples were not used for training). **C** PCA plot presents a separation between SCZ patients (green) and healthy individuals (blue). **D** Feature Importance plot of the features of the model. Feature importance is defined as the percentage drop in predictive performance when the feature is removed from the model. Abbreviations: ROC Receiver Operating Characteristic, PCA Principal Component Analysis.

To examine the potential role of the protein products of the genes featured in the GSE41037 and GSE157252 dataset biosignatures in the pathophysiology of SCZ, we utilized the literature mining tool *multiple* UniReD to assess functional associations between proteins, as previously applied [33]. For this analysis, we used a list of 33 protein-coding genes with a known role in SCZ according to literature (cf. Supplementary Table S1). All genes were found to be associated with SCZ pathways. In particular, *IGF2BP1* reached a score of 23, *MDGA1* reached a score of 15, *CENPI* reached a score of 10, *CISD3* reached a score of 9, and *PSME4* a score of 2 (Fig. 3).

In addition, we performed a functional protein association network analysis among IGF2BP1, PSME4, CENPI, MDGA1, CISD3, and the 33 protein-coding genes with a known role in SCZ used in the *multiple* UniReD analysis, and only CENPI displayed an association with UFD1L (cf. Supplementary Fig. S1).

### Laboratory methylation analyses in SCZ patients and CTRL individuals

**Fig. 4:**
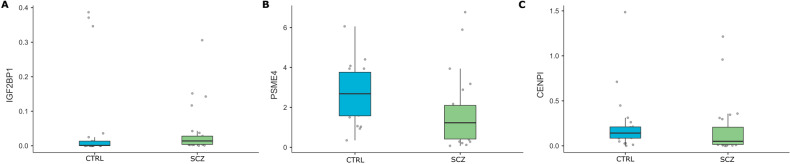
Methylation levels of *IGF2BP1, PSME4*, and *CENPI* between SCZ patients (green) and healthy individuals (CTRL) (blue). Methylation levels of (**A**) *IGF2BP1*, (**B**) *PSME4*, and (**C**) *CENPI* were estimated by qMSP in blood-derived gDNA from 30 drug-naïve SCZ patients (SCZ group) and 30 healthy individuals (CTRL group).

Following the in silico analysis, the methylation profiles of *IGF2BP1, PSME4*, and *CENPI* genes were investigated in SCZ patients compared to CTRL, to validate the biomarkers’ performance in discriminating SCZ in a clinical setting. Methylation levels of *IGF2BP1* and *PSME4*, but not *CENPI* were found to differ in a statistically significant way, *IGF2BP1* being higher and *PSME4* lower in the SCZ group as compared to the CTRL group (IGF2BP1: *U* = 156, nSCZ = nCTRL, *p* = 0.007, PSME4: *U* = 123, nSCZ = nCTRL, *p* = 0.015, CENPI: *U* = 196, nSCZ = nCTRL, *p* = 0.138). (cf. Fig. 4). Similarly, when groups were split according to sex, again methylation levels of *IGF2BP1* and *PSME4*, but not *CENPI* were found to differ in a statistically significant way, *IGF2BP1* being higher and *PSME4* lower in the SCZ female group as compared to the CTRL female group (IGF2BP1: *U* = 15, nSCZ = nCTRL, *p* = 0.001, PSME4: *U* = 12, nSCZ = nCTRL, *p* = 0.001, CENPI: *U* = 47, nSCZ = nCTRL, *p* = 0.160). No statistically significant differences were noticed between the male groups (cf. Supplementary Table S4). Also, no statistically significant differences were noticed between methylation levels of *IG2BP1, PSME4*, and *CENPI* and age, or any other clinical/demographical data (cf. Supplementary Table S4).

### Performance of single biomarkers in the diagnosis of schizophrenia

Among the three featured GSE41037 dataset biosignature genes, *IGF2BP1* showed the best AUC in the diagnosis of SCZ (AUC = 0.718) while *CENPI* and *PSME4* showed low AUC (AUC = 0.367 and AUC = 0.340, respectively). ROC curves are presented in Suppl. Figure S3.

### AutoML predictive analysis in the diagnosis of schizophrenia

**Fig. 5:**
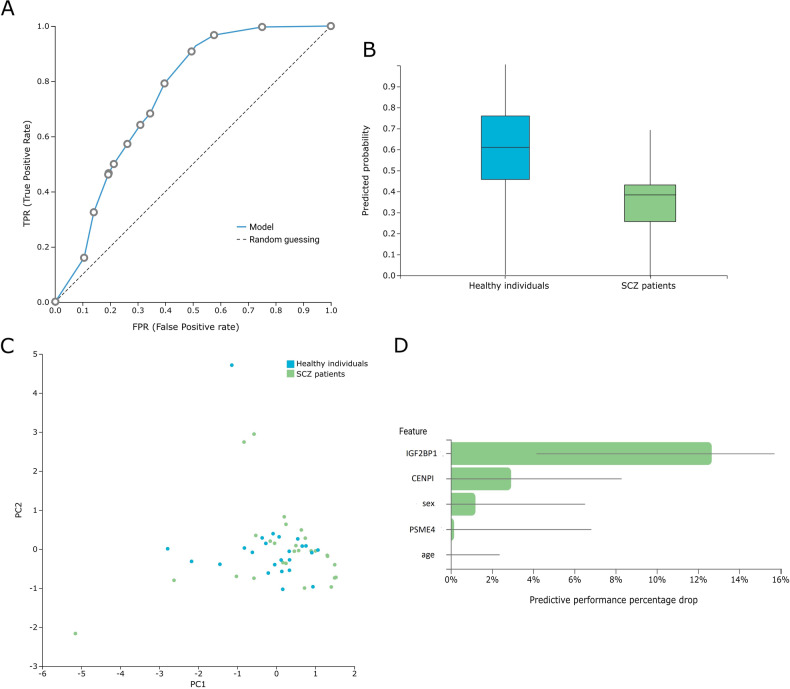
SCZ-specific methylation biosignature built from experimental methylation measurements using AutoML. **A** ROC curve of the model. **B** Out-of-sample probability box plot (i.e., probability predictions when samples were not used for training). **C** PCA plot shows the separation between SCZ patients at baseline (green) and healthy individuals (blue). **D** Feature Importance plot of the features of the model. Feature importance is defined as the percentage drop in predictive performance when the feature is removed from the model. Abbreviations: ROC Receiver Operating Characteristic, PCA Principal Component Analysis.

Our experimental data were further analyzed by the JADBio AutoML platform ML to produce diagnostic biosignatures validated in clinical samples. In our AutoML classification analysis, the task was to predict SCZ versus health combining the blood methylation measurements with demographic data. In this AutoML analysis, JADBio trained 3017 different machine learning pipelines (also called configurations), corresponding to different model types. Each one was employed many times during cross-validation (a repeated 10-fold CV without dropping, max. repeats = 20), leading to fitting 241,360 model instances (https://app.jadbio.com/share/5a59c593-9b99-48dc-9131-0447641ea556). The AutoML analysis produced a best-performing five-feature biosignature via the Classification Random Forests algorithm including methylation status of all three genes, age, and sex was able to discriminate groups with an AUC of 0.755 (0.636, 0.862), an accuracy of 0.720 (0.658,0,779) and an average precision of 0.758 (0.690, 0.825) (cf. Fig. 5). The methylation biomarkers were shown to bear higher importance into the model’s performance than age and gender as depicted by the feature Importance plot (Fig. 5D). In another AutoML Classification analysis where only age and sex were used as possible features produced biosignature resulted in an AUC of 0.545 (0.399, 0.686) bearing no classification power.

## Discussion

In the management of SCZ, there is a long-recognized need for new objective biomarkers that can improve diagnostic accuracy. In the present study, we introduce an AutoML-based pipeline that can identify disease-specific epigenetic biomarkers through feature selection and deliver highly performing methylation-based biosignatures with the aim of aiding the development of clinical tools for the accurate diagnosis of SCZ in blood. Therefore, as a first step, we analyzed in silico publicly available, high-throughput microarray blood methylation datasets from SCZ patients and healthy individuals through AutoML predictive analysis. Feature selection of AutoML analysis revealed three SCZ-specific methylation CpGs in *IGF2BP1, CENPI*, and *PSME4 genes* that combined in a model can predict SCZ in the methylome database. The methylation profile of the identified genes was then analyzed, in step 2, by targeted qMSP assays in blood gDNA of 30 first-episode SCZ patients and 30 healthy controls (CTRL). Finally, at step 3, AutoML analysis of our experimental clinical data combined with demographics led to a best-performing five-feature biosignature including all three genes, age, and sex, that was able to discriminate drug-naïve SCZ patients from healthy individuals with high AUC and precision. It is known and empirically expected that SCZ occurs more frequently in males and that young adulthood is the typical age of onset for SCZ. Therefore, age and sex were expected as potential predictive factors in our ML model. Still, the methylation biomarkers were indicated to bear higher importance in the model’s performance.

UniReD analysis was performed in order to reveal if AutoML-identified genes were somewhat associated with SCZ. It is important to mention, that while STRING is a standard bioinformatic tool used to identify possible protein-protein interaction networks by integrating information from various sources (literature, experiments, databases and genome context), *multiple* UniReD used here as an additional approach, is a novel computational tool which is able to not only identify known associations between proteins described in the biomedical literature, but also to predict novel interactions which are not yet experimentally documented. While STRING analysis revealed scarce associations, UniReD results demonstrated some relevance, especially *IGF2BP1* which showed the highest score when analyzed against a list of genes with an established role in SCZ pathophysiology. Indeed, IGF2BP’s family, including *IGF2BP1*, are key regulators of neuronal development, neuronal cell migration, and specification [55] being implicated in cytoskeletal signaling, translational control, Wnt/Hedgehog/Notch pathways [56, 57]. *IGF2BP1* and *CENPI* expression have been also found to be closely related to abnormal psychomotor behavior in SCZ [58]. In addition, *CENPI* expression was found to be significantly dysregulated between unaffected biological siblings and affected SCZ individuals [59]. Our approach, employing a data-driven unsupervised way to build classifying biosignatures, pointed to these three genes as bearing in combination high classifying power. Therefore, their involvement into SCZ pathophysiological pathways is worth further attention for a deeper understanding of SCZ biology, through functional in vitro or in vivo studies.

Previous studies leveraged either biological entities such as gut microbiota, blood gene expression, methylation, and SNPs, or neuroimaging and electroencephalogram data, and employed ML tools for SCZ discrimination with very promising results [60–65]. The predictive abilities of the models built were in the range of AUC 0.780 to 0.993. Chan et al. developed via LASSO regression method a serum protein-based biosignature of 26 biomarkers that discriminated efficiently first-onset, drug-naive SCZ patients from controls, reaching an AUC of 0.970 [60]. Lin et al. combined G72 genetic variation and its protein levels and developed a naive Bayes-based biosignature using G72 rs1421292 and G72 protein for identifying SCZ with high discriminative power (AUC = 0.935) [61]. Trakadis et al. using whole exome sequencing data developed a biosignature of 372 genes via the XGBoost algorithm that can predict efficiently individuals at high risk for SCZ (AUC = 0.950) [62]. In the study of Chen et al., an epigenetic signature based on blood DNA methylation data was built that differentiated SCZ from healthy control and other neurological disorders such as bipolar disorder (AUC = 0.780) [63]. Ke et al. developed a biosignature via support vector machines without feature selection and as input features multi-biological data. Among them, the top 5% discriminative features included gut microbiota features (Lactobacillus, Haemophilus, and Prevotella), blood features (superoxide dismutase level, monocyte-lymphocyte ratio, and neutrophil count), and the electroencephalogram features (nodal local efficiency, nodal efficiency, and nodal shortest path length in the temporal and frontal-parietal brain areas). This biosignature showed also high discriminative power (AUC = 0.970) [64]. Zhu et al. built an expression-based biosignature via SVM including the expression profile of six genes (*GNAI1, FYN, PRKCA, YWHAZ, PRKCB*, and *LYN*) that can differentiate SCZ patients from healthy individuals with the highest discriminative power so far (AUC = 0.993) [65]. More recently, a blood-based machine learning case-control classifier using DNA methylation data, by applying sparse partial least squares discriminating analysis on Human Methylation 450 K array data, demonstrated an AUC of 0.67 in discriminating SCZ from healthy individuals [66]. In addition, treatment-resistance SCZ was identified from non-resistant disease with a high accuracy of 88.3%, by a risk score model based on the blood methylation of 5 genes (LOC404266, *LOXL2, CERK, CHMP7*, and *SLC17A9*) identified by a ML algorithm applied on genome-wide methylation dataset analysis [67].

Here, in order to build methylation-based biosignatures, we employed, for the first time, AutoML using the JADBio platform. As we previously proposed [32, 34], this approach presents two major advantages for further developments in biomarker discovery. (a) It produces high-performing classifiers with low-feature numbers via feature selection, i.e., automatic calculations for identifying the minimum feature number within a dataset of some thousands of features that retain the maximum classifying power. (b) It has been shown to shield against typical methodological pitfalls in data analysis that lead to overfitting and overestimating performance and, therefore, to misleading results and in particular in low number biomedical datasets. The performance in terms of sensitivity/specificity of our model as shown in laboratory validation, is comparable to this reported for other models so far. Still, the low number of features in our model, the minimally-invasive approach along with the relatively simpler qPCR technology are significant advantages for a potential diagnostic test to be implemented in clinical practice. Reducing the feature size of a signature by feature selection via AutoML, is a great advantage towards more cost-effective assays with less technical requirements for multiplexing, moving from the multi-dimensional omics results to simpler classifiers. Furthermore, the AutoML-aided approach chosen here, has a proven capacity to refrain from performance overestimations, which is the main observed concern in transferring data-driven built models into real life. Indeed, JADBio has been shown to shield against typical methodological pitfalls in data analysis that lead to overfitting and overestimating performance and, therefore, to misleading results and in particular in low number biomedical datasets. It was shown that, on typical biomedical datasets, JADBio identifies signatures with just a handful of molecular quantities, while maintaining competitive predictive performance. Furthermore, an advancement of the use of machine learning classification tools, like JADBio is that those features that do not demonstrate statistically significant changes between groups (possibly due to the small group of patients), as in our study the *CENPI* gene methylation status, may be selected in a model together with other features, to add to a combined classification performance. At the same time, it reliably estimates the performance of the models from the training data alone, without losing samples to validation [24, 25]. Upon further extensive analytical and clinical validation, the signatures built can offer feasible solutions for laboratory tests that could be applied in a standardly equipped diagnostic lab.

Epigenetic modifications, including DNA methylation patterns, play an important role in regulating gene expression. These modifications can affect gene expression by promoting or inhibiting the binding of transcription factors and other regulatory proteins to DNA. Environmental factors such as diet, stress, pathogens, toxins, and lifestyle have been shown to trigger epigenetic changes in an exposure- and/or a disease-related manner. A combination of genetic and environmental risk factors is thought to influence the normal processes of brain development and maturation, manifesting as a range of neurotransmitter and circuit disorders and connectivity disorders in early adulthood, like SCZ [68]. In SCZ and other multifactorial neuropsychiatric diseases, epigenetic processes have been shown to mediate the effects of environmental risks, but may also interact with the genomic risks associated with these diseases [69]. In specific, there is evidence that prenatal exposure to certain environmental factors, such as maternal obstetric complications [70], malnutrition [71], or infections [72], has been associated with an increased risk of SCZ. Also, urban upbringing [73], cannabis use during adolescence [74], and childhood adversity [75] are also considered environmental risk factors for SCZ. Understanding environmental risk factors can enhance the predictive power of models assessing schizophrenia risk. Including variables related to prenatal or childhood exposures, psychosocial stressors, or substance use can improve the accuracy of risk prediction models. Future research should aim to integrate genetic, epigenetic, and environmental factors to provide a more comprehensive understanding of the complex interplay contributing to the development of schizophrenia. This approach will contribute to the development of more effective prevention and intervention strategies.

Changes in blood cell composition can influence DNA methylation patterns. Blood is a heterogeneous tissue composed of various cell types, including white blood cells (leukocytes), red blood cells, and platelets. Each cell type has a distinct DNA methylation profile, and alterations in the proportion of different cell types within the blood can contribute to changes in overall DNA methylation patterns. Previous studies have shown that SCZ is associated with elevated levels of WBC count (i.e., higher WBC count, lymphocyte count, neutrophil count, basophil count, eosinophil count, and monocyte count) [76]. Possibly, changes in blood cell composition can explain the DNA methylation alterations found in this study.

The relatively small group size of SCZ patients and healthy individuals participating in our experimental validation part of the study represents a limitation and possibly prohibits further significant differences in *IGF2BP1, CENPI*, and *PSME4* methylation levels to emerge. Nevertheless, although the sample size is one of many important design elements contributing to the successful implementation in biomarker discovery, the use of AutoML overcomes such limitations and aids robust and maximal data extrapolation from small cohorts. Future validation of built biosignatures in a larger group of patients should be conducted in order to confirm its clinical value and demonstrate performance in terms of sensitivity/specificity in the real-world setting. Further studies should also address the value of the methylation of each one of the identified genes, with both bioinformatic and experimental indications pointing to *IGF2BP1* as the one with the greatest interest. IGF2BP1, being a member of the IGF2BP family, is implicated in cytoskeletal signaling, translational control, Wnt/Hedgehog/Notch pathways, and regulation of cytokine production and neuronal development, migration and specification [56, 57]. PSME4 on the other hand is involved in the CDK-mediated phosphorylation and removal of Cdc6 pathway and DNA repair and cellular response to DNA damage stimulus [77]. As such, both genes deserve further attention through experimental approaches, aiming to unfold potential participation in SCZ pathogenetic processes, which could explain their value in discriminating SCZ patients. Identifying biomarkers that can be used as diagnostics or predictors of treatment response (theranostics) in people with SCZ will be an important step towards being able to provide personalized management of this complex mental disorder [67]. In addition, these biomarkers should also be tested against other diagnostic entities that share genetic overlaps with SCZ, such as bipolar disorder, depression, epilepsy, autism, and multiple sclerosis [78, 79].

Blood-based methylation biomarkers could offer a minimally invasive approach to early diagnosis and prediction of treatment response upon prospective evaluation [66, 67]. Our study demonstrates the potential of AutoML-driven data approaches in discovering novel blood-based epigenetic biomarkers in SCZ while also informing disease biology.

### Supplementary information

supplemental material

## Data Availability

The data utilized in the in silico part of the study are publicly available and the clinical data are available upon reasonable request.
