# Supplementary material for: A novel blood-based epigenetic biosignature in first-episode schizophrenia patients through automated machine learning
Source: Transl Psychiatry. 2024 Jun 17;14:257. doi: 10.1038/s41398-024-02946-4 (PMC11183091; doi:10.1038/s41398-024-02946-4)
Supplement: Supplementary file 1 — supplemental material [file 41398_2024_2946_MOESM1_ESM.docx]

# **SUPPLEMENTARY MATERIAL**

**Suppl. Table S1. Reference genes related to Schizophrenia used in multi-UniRed analysis.**

| **Gene** | **UniProt accession number** | **Reference** | **mUniRed score for *IGF2BP1*** | **mUniRed score for *PSME4*** | **mUniRed score for *CENPI*** |
| --- | --- | --- | --- | --- | --- |
| NRG1 | Q02297 | (1) | 1 | 0 | 0 |
| COMT | P21964 | (2) | 0.5 | 0 | 0 |
| DTNBP1 | Q96EV8 | (3) | 0.5 | 0 | 0 |
| RGS-4 | P49798 | (4) | 1 | 0 | 0.5 |
| 5HT2A | P28223 | (5) | 0.5 | 0 | 0 |
| 5HT1A | P08908 | (5) | 0.5 | 0 | 0 |
| DRD1 | P21728 | (6) | 0.5 | 0 | 0 |
| DRD2 | P14416 | (6) | 0.5 | 0 | 0 |
| DRD3 | P35462 | (7) | 0.5 | 0 | 0 |
| DRD4 | P21917 | (6) | 0 | 0 | 0 |
| DRD5 | P21918 | (6) | 0 | 0 | 0 |
| NDEL1 | Q9GZM8 | (8) | 0 | 0 | 1 |
| CRY1 | Q16526 | (9) | 0.5 | 0 | 1 |
| CRY2 | Q491N0 | (9) | 1 | 0 | 1 |
| RXRA | P19793 | (10) | 1 | 0 | 0.5 |
| PER2 | O15055 | (11) | 0.5 | 0 | 0.5 |
| ARNTL | O00327 | (11) | 1 | 1 | 1 |
| MBP | P02686 | (12) | 0.5 | 0 | 0 |
| PRG2 | P13727 | (13) | 0.5 | 0 | 0 |
| RELN | P78509 | (14) | 1 | 0 | 0 |
| AKT1 | P31749 | (15) | 1 | 1 | 1 |
| UFD1 | Q92890 | (16) | 1 | 0 | 0.5 |
| CLOCK | O15516 | (9) | 1 | 0 | 1 |
| PER1 | O15534 | (9) | 1 | 0 | 0.5 |
| PER3 | P56645 | (9) | 1 | 0 | 1 |
| GAD1 | Q99259 | (17) | 0.5 | 0 | 0 |
| RPS25 | P62851 | (18) | 0.5 | 0 | 0 |
| SLC6A4 | P31645 | (19) | 1 | 0 | 0 |
| BDNF | P23560 | (20) | 0 | 0 | 0 |
| DGCR8 | Q8WYQ5 | (21) | 1 | 0 | 0 |
| BMAL1 | P21145 | (9) | 1 | 0 | 0 |
| MAP2K1 | Q02750 | (22) | 1 | 0 | 0.5 |
| DISC1 | Q9NRI5 | (23) | 0.5 | 0 | 0 |

**Suppl. Table S2. Demographic and clinical data of study groups.**

| **Demographics & Psychometrics** | **SCZ** | **CTRL** |
| --- | --- | --- |
| **Sex (female)** | 12 (40%) | 13 (43%) |
| **Age (years)** | 32 ± 9 | 30 ± 8 |
| **Family history (free)** | 21 (70%) | 30 (100%) |
| **Positive Scale Score (PANSS)** | 24 ± 6 | N/A |
| **Negative Scale Score (PANSS)** | 24 ± 6 | N/A |
| **General Pathology Score (PANSS)** | 45 ± 9 | N/A |

**Table Legend:**

Sex and family history are presented in absolute numbers (percentage). Age and psychometric scores are presented in means ± SD. SCZ: schizophrenia patients; CTRL: healthy controls; PANSS: positive and negative syndrome scale; N/A: non-applicable.

**Suppl. Table S3. Primer sequences and their genomic locations used for qMSP assays.**

| **Primer** | **Primer Sequence (5’ -3’)** | **Genomic location** |
| --- | --- | --- |
| ACTB-F | TGGTGATGGAGGAGGTTTAGTAAGT | 7:5,558,705:-1 |
| ACTB-R | AACCAATAAAACCTACTCCTCCC | 7:5,558,838:-1 |
| IGF2BP1-F | CGGATTTGGAGAAAGTGTTTGCGGAG | 17: 48,997,792:1 |
| IGF2BP1-R | TCGTCCGAACAATCCACGAAAACG | 17:48,997,862:1 |
| CENPI-F | GAGTTATTACGTTCGGTCGAG | X:101,101,105:1 |
| CENPI-R | ATACAAACAACATATACAAATTCTCTC | X:101,101,169:1 |
| PSME4-F | TTTACGCGGAGCGGTTAGAC | 2:53,864,393:-1 |
| PSME4-R | CCCCCGAACCACAACTCT | 2:53,864,455:-1 |

**Suppl. Table S4.** **Methylation levels of IG2BP1, PSME4, and CENPI in study groups.**

|  |  | SCZ | CTRL | p-value |
| --- | --- | --- | --- | --- |
| *IGF2BP1* | *Total sample* | 0.016  (0 – 0.306) | 0.001  (0 – 0.387) | 0.007 |
|  | *Males* | 0.012  (0 - 0.306) | 0.007  (0 – 0.387) | 0.390 |
|  | *Females* | 0.021  (0 – 0.143) | 0.001  (0 - 0.371) | 0.001 |
| *PSME4* | *Total sample* | 1.232  (0 – 6.500) | 2.690  (0.351 - 6.060) | 0.015 |
|  | *Males* | 1.890  (0 – 6.500) | 1.774  (0.940 - 2.770) | 0.390 |
|  | *Females* | 0.946  (0 – 2.888) | 3.510  (0.351 - 6.060) | 0.001 |
| *CENPI* | *Total sample* | 0.052  (0 – 1.214) | 0.142  (0 – 1.485) | 0.138 |
|  | *Males* | 0.052  (0 – 0.310) | 0.066  (0 – 0.712) | 0.537 |
|  | *Females* | 0.086  (0 - 1.214) | 0.196  (0.089 – 1.485) | 0.160 |

**Table Legend:**

Methylation levels are given in median (minimum - maximum). SCZ: schizophrenia patients; CTRL: healthy controls.

**Suppl. Table S5. Methylation levels of IG2BP1, PSME4, and CENPI in in relation to demographic and clinical data.**

|  | SCZ | | | CTRL | | | p-value |
| --- | --- | --- | --- | --- | --- | --- | --- |
|  | ***IGF2BP1*** | ***PSME4*** | ***CENPI*** | ***IGF2BP1*** | ***PSME4*** | ***CENPI*** |  |
| Age (years) |  |  |  |  |  |  |  |
| < 31 years | 0.014  (0 – 0.152) | 1.093  (0.075 - 6.774) | 0.019  (0 - 1.214) | 0.001  (0 – 0.371) | 2.274  (0.940 – 6.063) | 0.133  (0.029 – 0.448) | 0.926^#^  0.912^$^  0.123^&^ |
| ≥ 31 years | 0.017  (0 – 0.360) | 1.059  (0.121 – 3.945) | 0.111  (0.032 – 0.358) | 0.001  (0 – 0.387) | 2.936  (0.351 – 4.408) | 0.148  (0.001 - 1.485) |  |
| Family history |  |  |  |  |  |  |  |
| positive | 0.011  (0.001 - 0.037) | 0.488  (0.075 – 1.485) | 0.028  (0.001 - 0.358) |  |  |  | 0.356^#^  0.115^$^  0.526^&^ |
| negative | 0.016  (0 - 0.306) | 1.504  (0.121 – 6.500) | 0.067  (0 – 1.214) |  |  |  |  |
| Positive Scale Score (PANSS) |  |  |  |  |  |  |  |
| < 24 | 0.023  (0.003 - 0.306) | 1.232  (0.287 - 6.500) | 0.049  (0.009 – 1.214) |  |  |  | 0.278^#^  0.521^$^  0.616^&^ |
| ≥ 24 | 0.014  (0 - 0.152) | 1.116  (0.075 – 5.897) | 0.082  (0 – 0.346) |  |  |  |  |
| Negative Scale Score (PANSS) |  |  |  |  |  |  |  |
| < 24 | 0.011  (0 - 0.117) | 1.232  (0.075 - 6.500) | 0.065  (0.001 – 1.214) |  |  |  | 0.109^#^  1.000^$^  1.000^&^ |
| ≥ 24 | 0.023  (0.004 - 0.306) | 1.393  (0.212 – 3.945) | 0.095  (0 – 0.959) |  |  |  |  |
| General Pathology Score (PANSS) |  |  |  |  |  |  |  |
| < 45 | 0.015  (0 - 0.306) | 1.338  (0.075 – 3.182) | 0.134  (0.001 – 1.214) |  |  |  | 0.620^#^  0.941^$^  0.333^&^ |
| ≥ 45 | 0.020  (0.003 - 0.152) | 0.895  (0.212 – 6.500) | 0.049  (0 – 0.959) |  |  |  |  |

**Table Legend:**

Methylation levels are given in median (minimum - maximum). SCZ: schizophrenia patients; CTRL: healthy controls. # indicates p-value for *IGF2BP1,* $ indicates p-value for *PSME4*, and & indicates p-value for *CENPI*.

**Suppl. Figure S1. Protein-protein interaction networks among** **IGF2BP1, PSME4, CENPI, MDGA1, CISD3 and the 33 protein-coding genes with a known role in SCZ.**


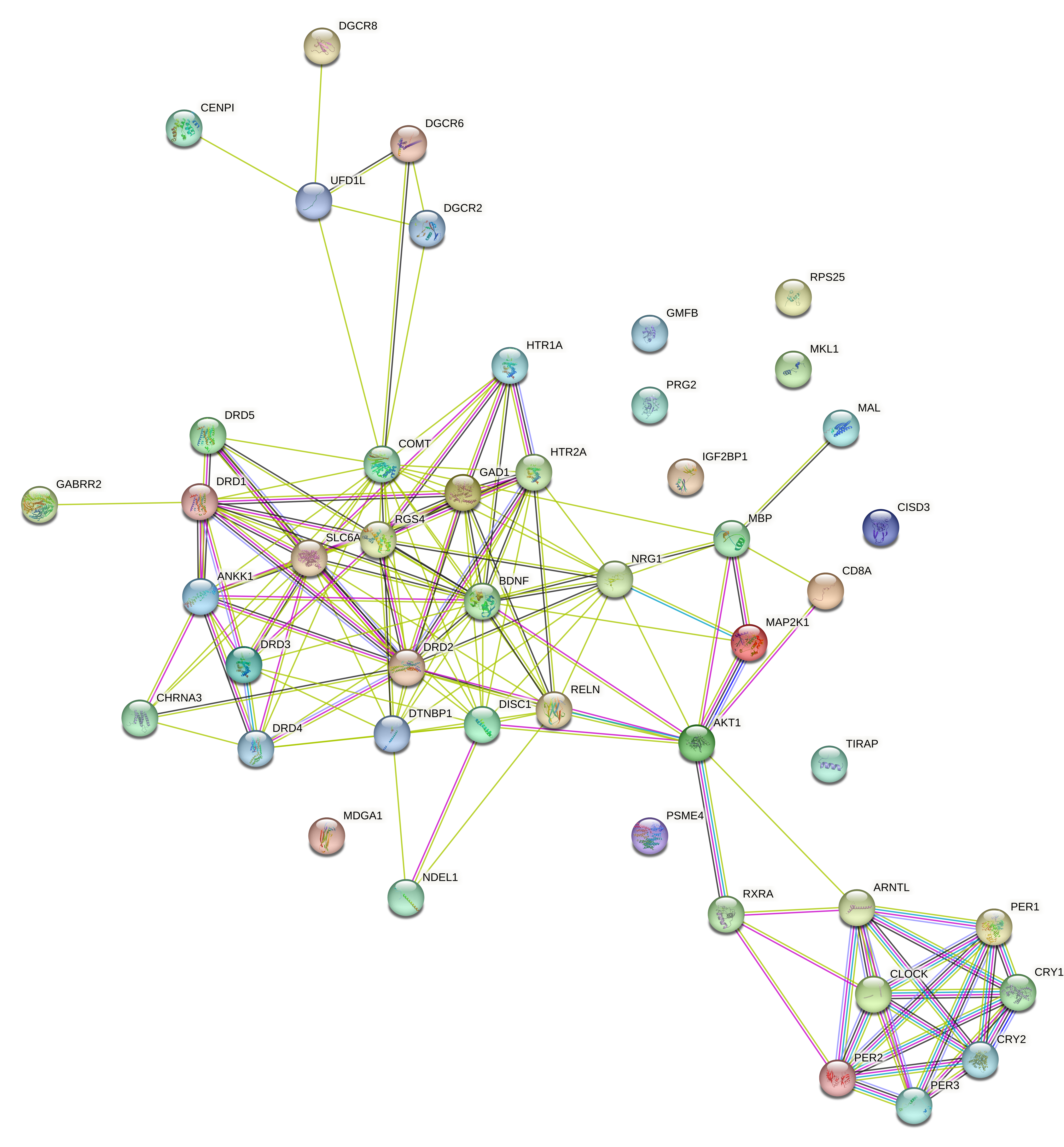


**Figure Legend:**

A functional protein association network analysis among IGF2BP1, PSME4, CENPI, MDGA1, CISD3 and the 33 protein-coding genes using the STRING database, a database of known and predicted protein-protein interactions. Only CENPI showed a protein-protein interaction with UFD1L.

**Suppl. Figure S3. ROC curve analysis results discriminating SCZ patients and CTRL.**


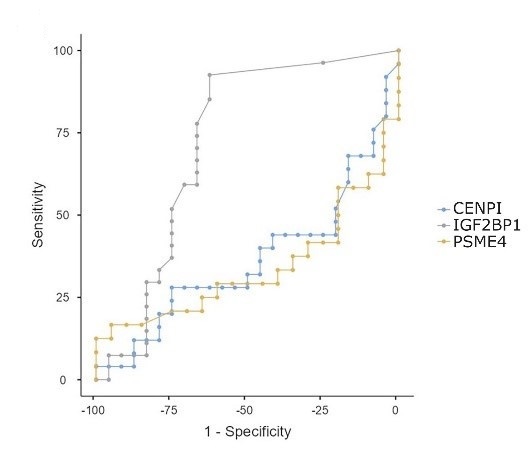


**Figure Legend:**

ROC curves of *CENPI, IGF2BP1*, and *PSME4* discriminating SCZ patients and healthy individuals.

## **References**

1. Stefansson H, Sigurdsson E, Steinthorsdottir V, Bjornsdottir S, Sigmundsson T, Ghosh S *et al.* Neuregulin 1 and susceptibility to schizophrenia. *American journal of human genetics* 2002; **71**(4)**:** 877-892.

2. Shifman S, Bronstein M, Sternfeld M, Pisanté-Shalom A, Lev-Lehman E, Weizman A *et al.* A Highly Significant Association between a COMT Haplotype and Schizophrenia. *The American Journal of Human Genetics* 2002; **71**(6)**:** 1296-1302.

3. Van Den Bogaert A, Schumacher J, Schulze TG, Otte AC, Ohlraun S, Kovalenko S *et al.* The DTNBP1 (dysbindin) gene contributes to schizophrenia, depending on family history of the disease. *American journal of human genetics* 2003; **73**(6)**:** 1438-1443.

4. Erdely HA, Tamminga CA, Roberts RC, Vogel MW. Regional alterations in RGS4 protein in schizophrenia. *Synapse (New York, NY)* 2006; **59**(8)**:** 472-479.

5. Meltzer HY, Li Z, Huang M, Prus A. Serotonergic mechanisms in schizophrenia: Evolution and current concepts. *Current Psychosis & Therapeutics Reports* 2006; **4**(1)**:** 12-19.

6. Ma L, Zhang X, Xiang Q, Zhou S, Zhao N, Xie Q *et al.* Association between dopamine receptor gene polymorphisms and effects of risperidone treatment: A systematic review and meta-analysis. *Basic & Clinical Pharmacology & Toxicology* 2019; **124**(1)**:** 94-104.

7. Nunokawa A, Watanabe Y, Kaneko N, Sugai T, Yazaki S, Arinami T *et al.* The dopamine D3 receptor (DRD3) gene and risk of schizophrenia: case-control studies and an updated meta-analysis. *Schizophrenia research* 2010; **116**(1)**:** 61-67.

8. Dal Mas C, Nani JV, Noto C, Yonamine CM, da Cunha GR, Mansur RB *et al.* Ndel1 oligopeptidase activity as a potential biomarker of early stages of schizophrenia. *Schizophrenia research* 2019; **208:** 202-208.

9. Johansson AS, Owe-Larsson B, Hetta J, Lundkvist GB. Altered circadian clock gene expression in patients with schizophrenia. *Schizophrenia research* 2016; **174**(1-3)**:** 17-23.

10. Rioux L, Arnold SE. The expression of retinoic acid receptor alpha is increased in the granule cells of the dentate gyrus in schizophrenia. *Psychiatry research* 2005; **133**(1)**:** 13-21.

11. Liu JJ, Sudic Hukic D, Forsell Y, Schalling M, Ösby U, Lavebratt C. Depression-associated ARNTL and PER2 genetic variants in psychotic disorders. *Chronobiology international* 2015; **32**(4)**:** 579-584.

12. Ota VK, Noto C, Santoro ML, Spindola LM, Gouvea ES, Carvalho CM *et al.* Increased expression of NDEL1 and MBP genes in the peripheral blood of antipsychotic-naïve patients with first-episode psychosis. *European neuropsychopharmacology : the journal of the European College of Neuropsychopharmacology* 2015; **25**(12)**:** 2416-2425.

13. Cohen OS, McCoy SY, Middleton FA, Bialosuknia S, Zhang-James Y, Liu L *et al.* Transcriptomic analysis of postmortem brain identifies dysregulated splicing events in novel candidate genes for schizophrenia. *Schizophrenia research* 2012; **142**(1-3)**:** 188-199.

14. Ovadia G, Shifman S. The genetic variation of RELN expression in schizophrenia and bipolar disorder. *PloS one* 2011; **6**(5)**:** e19955.

15. Thiselton DL, Vladimirov VI, Kuo PH, McClay J, Wormley B, Fanous A *et al.* AKT1 is associated with schizophrenia across multiple symptom dimensions in the Irish study of high density schizophrenia families. *Biological psychiatry* 2008; **63**(5)**:** 449-457.

16. Ota VK, Berberian AA, Gadelha A, Santoro ML, Ottoni GL, Matsuzaka CT *et al.* Polymorphisms in schizophrenia candidate gene UFD1L may contribute to cognitive deficits. *Psychiatry research* 2013; **209**(1)**:** 110-113.

17. Akbarian S, Huang H-S. Molecular and cellular mechanisms of altered GAD1/GAD67 expression in schizophrenia and related disorders. *Brain Research Reviews* 2006; **52**(2)**:** 293-304.

18. Wagh VV, Vyas P, Agrawal S, Pachpor TA, Paralikar V, Khare SP. Peripheral Blood-Based Gene Expression Studies in Schizophrenia: A Systematic Review. *Front Genet* 2021; **12:** 736483.

19. Xu FL, Wang BJ, Yao J. Association between the SLC6A4 gene and schizophrenia: an updated meta-analysis. *Neuropsychiatric disease and treatment* 2019; **15:** 143-155.

20. Gören JL. Brain-derived neurotrophic factor and schizophrenia. *The mental health clinician* 2016; **6**(6)**:** 285-288.

21. Beveridge NJ, Gardiner E, Carroll AP, Tooney PA, Cairns MJ. Schizophrenia is associated with an increase in cortical microRNA biogenesis. *Molecular Psychiatry* 2010; **15**(12)**:** 1176-1189.

22. Wang J, Su P, Yang J, Xu L, Yuan A, Li C *et al.* The D2R-DISC1 protein complex and associated proteins are altered in schizophrenia and normalized with antipsychotic treatment. *Journal of psychiatry & neuroscience : JPN* 2022; **47**(2)**:** E134-e147.

23. Uzuneser TC, Speidel J, Kogias G, Wang A-L, de Souza Silva MA, Huston JP *et al.* Disrupted-in-Schizophrenia 1 (DISC1) Overexpression and Juvenile Immune Activation Cause Sex-Specific Schizophrenia-Related Psychopathology in Rats. *Front Psychiatry* 2019; **10:** 222.
